# Supplementary material for: The Kaposi’s Sarcoma-Associated Herpesvirus Protein ORF42 Is Required for Efficient Virion Production and Expression of Viral Proteins
Source: Viruses. 2019 Aug 2;11(8):711. doi: 10.3390/v11080711 (PMC6722526; doi:10.3390/v11080711)
Supplement: Supplementary file 1 [file viruses-11-00711-s001.pdf]

1	atgtccctggaaagggccctggcgagactgactggagtgccaatgagtactcatgccccgaagactcgcg	70
71	agtcagaagaggcctgtcccgtataccccaccctgtggtgccccgccttgtcctggaagtgcataggaa	140
141	aaacaacgcactggttggaagcaataccccaaaatgtccgtgatgggccacttagatatagcgtgtctc	210
211	agggagcacgtgaaatctcgcctgtcctcgcccaactttgtgggatttacggtggtgtgcctggtggagc	280
281	acgaagacatggtgacccacatagacctacaccctcacgtgttccaagagcgagtgtgcctccttagacc	350
351	tacatctcccggcataaccgagctctgctgcctgttatcgatgctcgagaactgtcgagacatgtcacca	420
421	acgtttttgagatctattatttgtcgcgccagaaagacacatgagaggacgccggggatggacgcggcct	490
491	tcgtcatgcacggtatagagacgttgactgccacggccgcctttgtttacgagctgtccgtggacgatca	560
561	tttcagggccaccccactcgttatgtttaagcttcataaggcgataggcgacgcttcaaccccccatgggg	630
631	ggactaatgaaacccatttatctagagtcttttaaactggagtctagtggagagaatgaag**atgacaaac**	700
701	**aaacctgcgcggacccagtaaacatttttattgtgataccatctttaccaagcacctggagaacaatga**	770
771	**agttttaaaatatctgaaaatgtgcacccttttcaaaccccccattgtttcccttttttcaaaat**caggg	840
841	cggccgctcgagggaggcggtggagccgactacaaggaccacgacggcgactacaaggaccacgacatcg	910
911	actacaaggacgacgacgacaaggggcccgtt*taa***atgacaaacaaacctgcgcggacccagtaaacatt**	980
981	**ttttattgtgataccatctttaccaagcacctggagaacaatgaagttttaaaatatctgaaaatgtgca**	1050
1051	**cccttttcaaaccccccattgtttcccttttttcaa*****aat*** *aaa*gataaaagcctggtccaggcctctagct	1120
1121	gtagagtcatttcctttgcgtcttggggggttgtgttcctgtgggctaacttgccggacagctttcagagt	1190
1191	ctgctctaaatagaacaggtaattaacatccacaggaagcgaaaagcctgcccggtaacacatgggtccg	1260
1261	gactttgttactataaacagccttggtgctaggcagtcaaatggtaaagggggcatactgggatccctt	1330
1331	cacagtgcgataggggatgagccagaatgatgtttgtcttcgcctatgaaaatacaggggtatcacttc	1400
1401	ctctatcagctgcagccaggaagtgtcgggaggcatgtttagggctcgttcaatgcatgtggttaagctt	1470
1471	tgcagccaggtaacaaagccgcagaggttgggggagtttctgtgttccccatttccctcagtgtctggtc	1540
1541	cggtgatgtgacgtgggttaggctcacccccgtccccgctgtcgaccttggactgcctgcatggaggaa	1610
1611	atatcccatcgaggttgaaaagagcaagttcgcatgcgatgagaatacaagatcccccgaagtccccccc	1680
1681	ttcagagtaaacccggccat	1700

Light blue	ORF42 coding sequence (including mutation in stop codon)
Orange	Flag tag
Green	ORF41 coding sequence (on opposite strand)
Underline and italics	stop codon and polyadenylation signals for ORF42
**Bold**	duplicated sequences (end of OR42 and 3' region)
**Underline and bold**	polydenylation signal for ORF41 (in duplicated region)
